# Preferences for coaching strategies in a personalized virtual coach for emotional eaters: an explorative study

**DOI:** 10.3389/fpsyg.2023.1260229

**Published:** 2023-11-16

**Authors:** Aranka Dol, Tatjana van Strien, Hugo Velthuijsen, Lisette van Gemert-Pijnen, Christina Bode

**Affiliations:** ^1^Department of Psychology, Health and Technology, University of Twente, Enschede, Netherlands; ^2^Institute for Communication, Media & IT, Hanze University, Groningen, Netherlands; ^3^Behavioural Science Institute, Radboud University, Nijmegen, Netherlands

**Keywords:** personalized virtual coach, coaching strategies, emotional eating, persona, vignette

## Abstract

**Objectives:**

Emotional eating is recognized as a potential contributor to weight gain. Emotional eaters often hide their problems because of feelings of shame about their behavior, making it challenging to provide them with the necessary support. The introduction of a virtual coach might offer a potential solution in assisting them. To find out whether emotional eaters are receptive to online personalized coaching, we presented emotional eaters with two essential proto-typical problem situations for emotional eaters: “experiencing cravings” and “after giving in to cravings,” and asked them whether they preferred one of the three coaching strategies presented: Validating, Focus-on-Change and Dialectical.

**Methods:**

An experimental vignette study (2 × 3 design) was carried out. The vignettes featured two distinct personas, each representing one of the two common problem scenarios experienced by emotional eaters, along with three distinct coaching strategies for each scenario. To identify potential predictors for recognition of problem situations, questionnaires on emotional eating (DEBQ), personality traits (Big-5), well-being (PANAS), and BMI were administrated.

**Results:**

A total of 62% of the respondents identified themselves with “after giving in to cravings” and 47% with “experiencing cravings.” BMI, emotional eating and emotional stability appeared to be predictors in recognizing both the problem situations. In “experiencing cravings,” the participating women preferred Dialectical and the Validation coaching strategies. In the “after giving in to cravings” condition, they revealed a preference for the Dialectical and the Focus-on-Change coaching strategies.

**Conclusion:**

Using vignettes allowed a less threatening way of bringing up sensitive topics for emotional eaters. The personas representing the problem situations were reasonably well recognized. To further enhance this recognition, it is important for the design and content of the personas to be even more closely related to the typical problem scenarios of emotional eaters, rather than focusing on physical characteristics or social backgrounds. This way, users may be less distracted by these factors. With the knowledge gained about the predictors that may influence recognition of the problem situations, design for coaching can be more customized. The participants represented individuals with high emotional eating levels, enhancing external validity.

## 1. Introduction

With 13 percent of the world’s adult population, 18 years and older, suffering from obesity^1^ and 39 percent meeting the criteria of being overweight (World Health Organization, n.d.), both health care and public health need to find additional approaches, such as virtual coaching systems, to combat this pandemic. Emotional eating is the tendency to overeat in response to negative emotions, and is considered a risk factor for weight gain, obesity and eating disorders (Wardle, 1987; McManus and Waller, 1995; Deaver et al., 2003; Sung et al., 2009; Koenders and Van Strien, 2011; Frayn and Knäuper, 2018; Van Strien, 2018; Reichenberger et al., 2020), and is seen as a key characteristic of overeating and binge eating. Emotional eating is an atypical stress response. A normal reaction to negative emotions, such as stress, is a loss of appetite, prompted by bodily reactions that prepare humans for a fight or flight response, suppressing appetite (Gold and Chrousos, 2002; Van Strien and Ouwens, 2007; Van Strien et al., 2012a).

Therefore, it is our aim to develop a personalized virtual coach that focuses on counteracting emotional eating, here defined as the urge to (over)eat in reaction to negative emotions (Nolan et al., 2010; Van Strien et al., 2012b). This virtual coach, yet to be developed, will be a smartphone application in which users can interact with a virtual caregiver.

Face-to-face therapy and counseling, such as personal coaching, personal feedback, and education, as provided in behavioral therapies such as Cognitive Behavior Therapy (CBT), and Dialectical Behavior Therapy (DBT), are effective means of helping the emotional eater (Telch et al., 2001; Glisenti and Strodl, 2012; Roosen et al., 2012; Frayn and Knäuper, 2018; Safer et al., 2018; Rozakou-Soumalia et al., 2021; Braden et al., 2022; Hany et al., 2022).

However, to prevent or reduce emotional eating behavior and getting adequate help in time to the recipient is not always easy. Firstly, the patient or client is often distanced from health care (because of shame or embarrassment). Emotional eaters will have difficulties contacting care providers on their own initiative. Secondly, therapists are not available when the need is most urgent for the emotional eater. We distinguish two proto-typical problem situations here 1) when experiencing cravings or 2) just after having given in to cravings (Safer et al., 2018; Dol et al., 2021).

Thirdly, health care is overburdened - the waiting lists are very long. There is too little specialist care available for this specific group of patients. And lastly, this patient group should have a greater presence in the public health system as emotional eating is a risk factor for weight gain and obesity.

In order to improve right-on-time coaching for emotional eaters, providing ehealth solutions may be a good addition to existing treatments (Baez et al., 2016; Hartanto et al., 2016; Salvi et al., 2018; van der Kolk et al., 2019; Prvu Bettger et al., 2020; Tsiouris et al., 2020). Digital technologies have demonstrated that they can provide user-friendly solutions in the capacity of smartphone applications that offer appropriate exercises or personalized content. A personalized virtual coach might assist the user by providing good advice, education, practical suggestions and motivational messaging, but also by tracking and analyzing inadequate behavior and the triggers prior to that behavior (by self-monitoring), and by offering appropriate exercises (Franklin et al., 2017; Oosterveen et al., 2017; Abrahamsson et al., 2018; Ramchandani, 2019; Bevilacqua et al., 2020; Kamali et al., 2020; Hurmuz et al., 2022). Coaching is optimal in a face-to-face situation (“gold standard”): it creates a reciprocal bond between client and caregiver. This promotes mutual trust and achieves quality in the caregiver-client relationship. Recently it has been shown that coaching does not necessarily have to be face-to-face to create a therapeutic alliance, a bond between therapist and client, but that online coaching has also shown the ability to do so (Sucala et al., 2012; Fernandez et al., 2021; Cilliers et al., 2022; Eichenberg et al., 2022).

Not only the timing and the 24/7 availability of coaching are important, the essence of the message and the tone of voice demand a good fit, adapted to the situation at hand (Safer et al., 2009; Rimondini, 2011; Pederson, 2015; Wenzel, 2017).

Successful therapies for treating emotional eating behavior such as Cognitive Behavior Therapy (CBT) (Fairburn et al., 2003; Ricca et al., 2010; Rimondini, 2011; Frayn and Knäuper, 2018) and Dialectical Behavior Therapy (DBT) (Wisniewski and Kelly, 2003; Safer et al., 2009; Bankoff et al., 2012; Roosen et al., 2012; Safer et al., 2018) employ a variety of communication strategies in the face-to-face treatment to put the client on the track to insight and the willingness to move to action. “DBT is an eclectic mix of concepts and techniques from a wide variety of psychological and philosophical approaches.” (Pederson, 2015, p23). It combines change-oriented interventions from cognitive behavioral therapy with acceptance techniques from Zen Buddhism (Linehan, 1993). The treatment program was adapted for Boulimia Nervosa and Binge Eating Disorder (BED) by Safer et al. (2009). Since BED is closely related to emotional eating behavior, this program is also tailored for emotional eaters (Roosen et al., 2012; Safer et al., 2018).

Face-to-face therapy is a fast-paced interaction between therapist and coachee, in which the therapist not only anticipates the coachee’s response, but can already predict, based on his facial expressions, tone of voice, and contextual information, what that response will be. Reality dictates that social intelligence and interaction at such a level are not (yet) possible for a personalized virtual coach (Wang et al., 2020).

As mentioned earlier a virtual coach can be experienced as impersonal (Tantam, 2006). The perceived interpersonal closeness is lacking, and the likelihood of dropout is greater than with face-to-face therapy (Kaltenthaler et al., 2008), but on the positive side, a personalized virtual coach is always available. It is able to provide right-on-time coaching, exactly at the difficult moments while “experiencing cravings” and “after giving in to cravings” when the human therapist is not available.

Virtual coaching based on DBT is still something of the future. Since the outbreak of COVID-19, therapists have begun to offer DBT online (van Leeuwen et al., 2021; Hyland et al., 2022), but the present studies involved remote therapy with the intervention of a therapist (telehealth, tele-DBT), delivered by video link or telephone.

In this study, we will assume 3 coaching strategies, inspired by DBT, focussing on two proto-typical problem situations:

1. Validation strategies suggest response from the coach in an empathic way, by hearing another person’s point of view and accepting them (and their emotions) without judging,
2. Focus-on-Change strategies present the receiver with a practical change-oriented focus on problem behavior by providing practical advise,
3. using Dialectical strategies the coach focusses on pairing of Validation and Focus-on-Change. The key here is finding a balance between acceptance of intense feelings and emotions and the need for change by adapting feelings and emotions.

To best meet the emotional eaters’ need for right-on-time coaching (Gravenhorst et al., 2015; Dekker et al., 2020), we have choosen the two tipping points, the two proto-typical problem situations (Safer et al., 2009; Dol et al., 2021), at which interventions might be most effective:

- “experiencing cravings,” when experiencing negative emotions and distress,
- “after giving in to cravings,” undergoing feelings of disappointment, anger, shame and disgust (Safer et al., 2018).

The two problem situations are depicted by personas. Personas are created by designers to act as “fictitious, specific and concrete representations of target users” (Pruitt and Adlin, 2010; Humphrey, 2017) “Personas are not real people, but they are based on the behaviors and motivations of real people we have observed and represent them throughout the design process” (Cooper et al., 2007, p75).

A persona acts as a prototype because it represents a group of users. It is constructed from characteristics of that target group based on data from the literature, and data from research among users (attributes related to emotional eating behavior). A persona is rich in composition by placing it in a context and by providing it with a personal profile, background, and user needs (LeRouge et al., 2013; Dol et al., 2016, 2017).

In the literature, the need for, or importance of, validation of personas is mainly associated with User Centered Design - to support the design of applications, for creating user stories, for heuristic evaluations, and to spur creativity among developers (Thoma and Williams, 2009; Pruitt and Adlin, 2010; Friess, 2015; Ferreira et al., 2018; Subrahmaniyan et al., 2018; Bonnardel and Pichot, 2020; Salminen et al., 2021).

Little has been published about the validation of personas used for experimental or exploratory research.

We chose to use personas because they may make the depiction of the proto-typical problem situations more insightful and accessible to the participants than presenting an abstract description, conducting interviews, or presenting mock-ups.

It is very important that participants and future users of the personalized virtual coach recognize themselves in one or both problem situations, because these two situations are the most essential situations for emotional eaters (Safer et al., 2009). It is expected that the virtual coach will be based on these problem situations, enabling the provision of appropriate coaching feedback. By presenting the problem situations we want to verify with the participants whether there is recognition in the two problem situations.

The personas presented to the participating women in this study, were Anita – representing the problem situation “experiencing cravings,” and Lisanne – representing “after giving in to cravings” see Table 1.

**Table 1:** Personas Anita and Lisanne, representing resp. problem situations “experiencing cravings,” and “after giving in to cravings.”

This is Anita 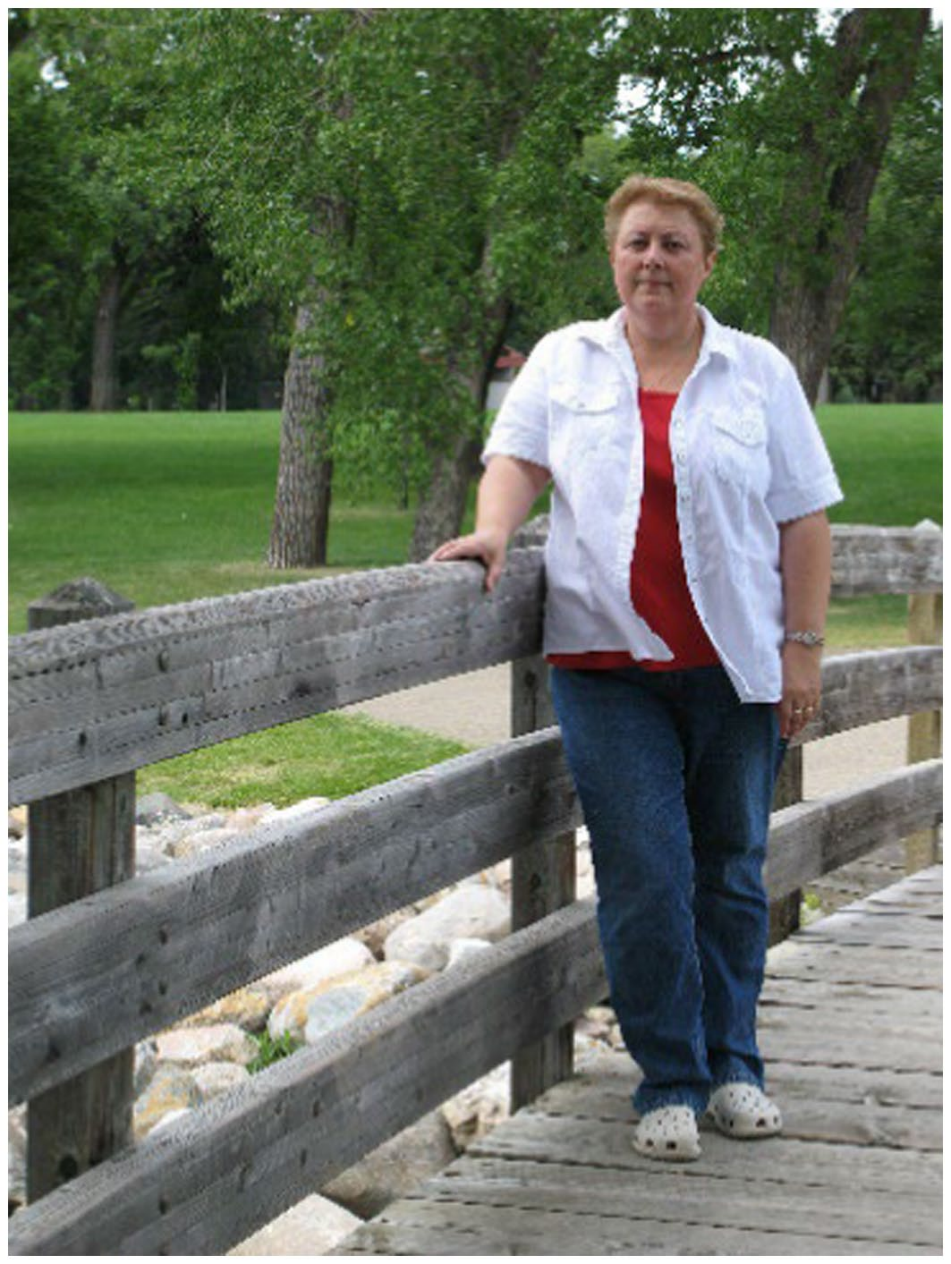	This is Lisanne 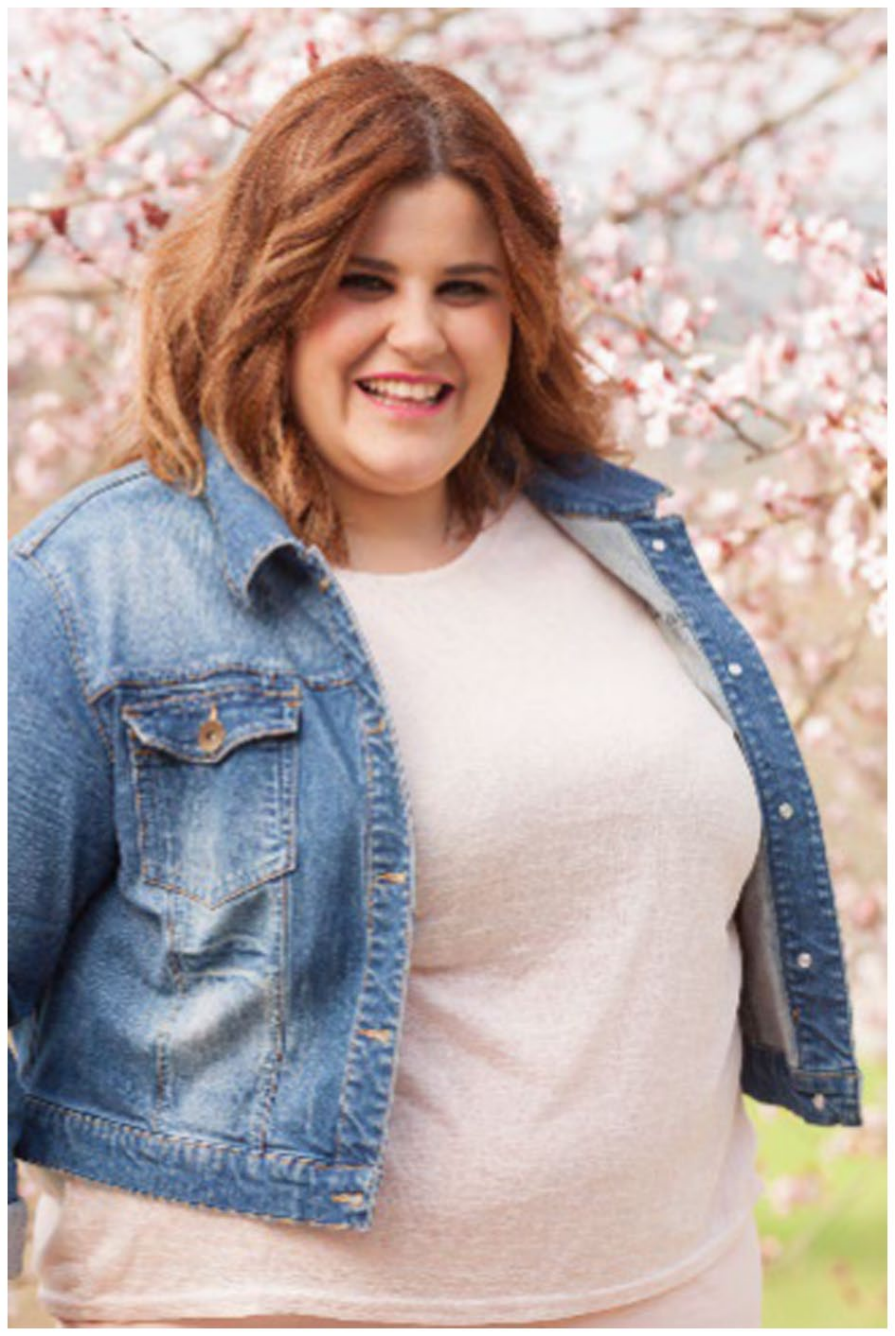
Anita is 46 years old. She is married to Ad They have two children, Melvin aged 19, and Leroy aged 16 Anita went to home economics school (LHNO) She works 3 days a week in home care. Other than that, she does housekeeping Her hobbies are playing games on the computer and hiking (if Ad at least comes along)	Lisanne is 25 years old. She is single, living alone. She has lived together with Peter for two years She has an MBO degree in commercial engineering and she works 5 days a week as an inside sales representative at a large real estate agency Her hobbies are going out, and shopping Sports: formerly korfball, but nothing at the moment
Anita weighs 85 kilograms. She gains a few kilos every year When tensions mount, Anita starts snacking “Food provides some kind of comfort for me. When I feel stressed, I’m always snacking. That’s really a problem, because because of this I’ve gained about 12 pounds in recent years”	Lisanne weighs 91 kilograms. She started dieting when she was 14 years old. Then she weighed 63 kilos. Lisanne feels insecure about her body She is unable to feel the difference between hunger, cravings or emotion. Once or twice a week she binges. Eating does relieve her for a while, but afterwards she feels disgusted with herself “I wish that I was more confident about myself …”

The full description of the personas and the description of the problem situations can be found in the Appendix. The photos we used to personify the personas were purchased from http://Shutterstock.com.

The sections of the personas (personal profile, attributes, user needs, and background) were developed based on the literature and data collected (LeRouge et al., 2013). They were further “dressed up” with data about living conditions such as family, education, interests. The personas served as a starting point for a concept design (Dol et al., 2016).

### 1.1. Aim of this study

In order to enable the provision of online personalized coaching and the appropriate coaching for any individual, the purpose of this study is firstly to explore whether respondents recognize themselves in the presented proto-typical problem situations “experiencing cravings” and “after giving in to cravings”, to ensure that the coach, yet to be developed, learns to present the right coaching, appropriate to the user’s situation and secondly, whether, or to what extent, that recognition is being affected by specific characteristics, such as a tendency towards emotional eating, personality traits, and the degree of well-being. If we can predict this, we will be able to further tailor the support of the virtual coach.

In order to provide with the most appropriate coaching for the given problem situations, it is important to determine which coaching strategy would be preferred by emotional eaters in relation to the two given problem situations.

The objective is to gain more knowledge about recognition of the given problem situations so that the virtual coach is enabled to deliver the right coaching based on this information. With this focus in mind, we formulated the following research questions:

*RQ1*: Are there predictors for identification with the typical problem situations for emotional eaters “experiencing cravings” and “after giving in to cravings”?

*RQ2*: What are the preferences for a specific coaching strategy with regards to the two typical problem situations for emotional eating?

## 2. Methods

### 2.1. Ethics

This study was approved by the ethical committee of the Faculty of Behavioral, Management and Social Sciences at the University of Twente (registration number 18033) on the 27th of February 2018, and was carried out between 13th of April and 13th of May 2018.

An information letter introduces the researcher and provides potential study participants with details about the study’s purpose, content, and the specific areas under investigation. The letter outlines the responsibilities expected from the participants, including providing personal information such as height, weight, and age, reading vignettes, answering related questions, and completing three questionnaires.

For reading the vignettes and answering the questions one online session is required. The estimated duration of the session is 45 min. Participants are also informed that the scenarios described in the study may be relatable or trigger strong empathetic reactions, which could potentially lead to personal reflection or discomfort. In light of this, informed consent is a critical aspect of the study.

### 2.2. Design

#### 2.2.1. Introduction study and personas

An experimental vignette study (2 × 3 design) was carried out. Vignettes may be used for three main purposes in social research: to allow actions in context to be explored; to clarify people’s judgments; and to provide a less personal and therefore less threatening way of exploring sensitive topics. The vignettes were used as a method of presenting the personas that depicted the two proto-typical problem situations for emotional eaters:

- “experiencing cravings,” when experiencing negative emotions and distress,
- “after giving in to cravings,” undergoing feelings of disappointment, anger, shame and disgust.

The vignettes subsequently produced one of three pre-defined coaching strategies: Validating, Focus-on-Change, and a Dialectical respons (Dol et al., 2021).

#### 2.2.2. Recruitment

To recruit participants, an invitation to participate was sent out to all dieticians of a particular franchise organization. They forwarded this request to their group of clients. A hyperlink in the email message directed the participants to an online survey (available on request). The clients were under treatment with the dieticians for weight reduction and lifestyle improvement. The dieticians asked their clients if they wanted to participate in a study on emotional eating. Those who answered affirmatively were forwarded the invitation to this study by e-mail.

#### 2.2.3. Participants

Adult women with self-proclaimed emotional eating difficulties (the mean score on emotional eating was M*_emo_* = 3,68; range 1,68-4,92) is highly similar to those of the norm-group of women with ages between 21 and 40 years (DEBQ); (weight M*_BMI_* = 30.61, range 18–45; and age M*_age_* = 44,9 range 20–70).

Participants were selected by the dieticians with whom they were under treatment. 109 respondents signed up in the Qualtrics online survey tool; 80 also started filling out a set of self-report instruments; eventually 62 completed all the questionnaires. It is not known why dropouts left the study before completion.

#### 2.2.4. Procedure

A pilot study was conducted among 6 participants to validate the study protocol. This pilot was performed with students at the faculty for Applied Psychology, at the Hanze University of Applied Sciences in Groningen. The actual research was carried out among participants who were recruited at a cooperative franchise organization of dieticians.

Participants received a login link for the Qualtrics online survey tool. They were presented with information on the study, followed by an online letter of consent they had to agree with, before proceeding to the questions. The participants filled in some demographic data, and thereafter, they were presented with two vignettes, followed by four questionnaires (see Measures).

They were invited to view the vignettes containing the presentation of the personas Lisanne and Anita (text and images as depicted in the Appendix). After the participants had read this, they could click through to the next screen with the coaching response (in text) from the so-called virtual coach. They were then presented with a series of questions (Supplementary Tables A3, A4). Finally, participants were subjected to a series of questionnaires to learn more about their personality, well-being, and affect in order to identify possible relationships between these characteristics and the extent to which they recognized a typical problem situation, and their preference for a coaching strategy.

#### 2.2.5. Measures

##### 2.2.5.1. Demographics

Participants shared information on their gender (they were all women), year of birth, length, and weight. Body mass index was calculated by dividing self-reported body weight (in kilogram) by height squared (in meters) [mass (kg) /height(m)^2^]. The average score of BMI is 30.61 (*N* = 67) (see Table 2). The year of birth was calculated into age in years [Age = (year of survey)−(year of birth)]. The average score of age is 44.90 (*N* = 78).

**Table 2:** Descriptives measures.

variable	*N*	Missing	Mean	Range	SD	Cronbach’s α
BMI	79	12	30.61	18.25–45.87	6.56	n.a.
Age	79	1	44.90	20–70	12.7	n.a.
Emotional eating	79	12	3.68	1.69–4.92	0.68	0.918 (13 items)
Openness	79	13	4.74	1.50–6.50	1.02	0.779 (6 items)
Conscientiousness	79	13	4.90	2.17–6.83	1.12	0.895 (6 items)
Extraversion	79	13	4.08	1.33–6.83	1.25	0.894 (6 items)
Agreeableness	79	13	5.69	3.67–6.83	0.66	0.803 (6 items)
Emotional stability (neuroticism)	79	13	3.80	1.67–5.67	1.08	0.803 (6 items)
PANAS pos	79	13	2.88	1.40–4.30	0.70	0.863 (10 items)
PANAS neg	79	13	1.78	1.00–3.90	0.68	0.860 (10 items)
PANAS SOM	79	13	0.62	0.37–0.78	0.10	n.a.

For the purpose of responding to RQ1, questionnaires on emotional eating behavior, personality traits and positive/negative affect were administered to participants. For the purpose of responding to RQ2 three questions were asked (see Supplementary Table A3).

##### 2.2.5.2. Questionnaire emotional eating behavior (DEBQ)

Emotional eating behavior was assessed using the Dutch Eating Behavior Questionnaire (DEBQ-E) (van Strien et al., 1986). The scale contains 33 questions about eating behavior, of which 13 items are about emotional eating. Each item was rated on a 5-point Likert scale ranging from 1 “never” to 5 “very often.”

##### 2.2.5.3. Questionnaire personality traits (quick big five)

The personality traits were assessed using the Quick Big Five Personality Questionnaire (Vermulst and Gerris, 2009). The scale contains 30 questions about personality traits. Each item was rated on a 7-point Likert scale ranging from 7 “is not true at all” to 1 “is absolutely right.”

##### 2.2.5.4. Questionnaire positive/negative affect (PANAS)

Positive and negative affect were measured (self-reported by participants) using the international Positive and Negative Affect Schedule (Watson et al., 1988). The Dutch translations of these emotions were derived from a Dutch version (Peeters et al., 1996).

The PANAS is a 20-item questionnaire that consists of 10 positive and 10 negative emotions. Each emotion was rated on a 5-point Likert scale ranging from 1 “very slightly” to 5 “very much.” The positive emotions are: alert, inspired, determined, attentive, active, enthusiastic, interested, exited, strong, and proud. The negative emotions are: scared, upset, distressed, jittery, guilty, irritable, hostile, ashamed, nervous, and afraid. The period over which the participants had to give their self-assessment was “at the present moment.”

The states of mind (SOM) represents the relative balance of positive and negative aspects of wellbeing. This model uses a ratio computed as P/(P + N) to define emotional and cognitive balance (Schwarz, 1986; Schwartz and Caramoni, 1989).

##### 2.2.5.5. Questions about the proto-typical problem situations and the coaching strategies

The participants were presented with three questions (multiple choice) about the vignettes (see Supplementary Table A3):

- one question (Q-1) about the problem situations “experiencing cravings” and “after giving in to cravings,”
- two questions (Q-2 and Q-3) about the presented coaching strategies (see Supplementary Table A4).

The answers were recoded into dichotomes to enable logistic regressions.

### 2.3. Statistical analyzes

All data were collected with qualtrics.com. Analyzes were conducted with SPSS statistical software, version 26 and Jamovi 2.2.5 (The Jamovi Project, 2021). The significance levels were set at 5%. Dichotomous variables were dummy coded as 0 or 1. Internal consistency was assessed with Cronbach’s Alpha (see Table 2), such as frequency, mean, standard deviation and range, were used to describe BMI, age, emotional eating, personality traits, positive and negative affect, and well-being, see Table 2.

To answer Research Question 1, contingency tables were performed, to test the relationship between recognition of the proto-typical problem situations and the type of coaching feedback. To compare the two problem situations “experiencing cravings” and “after giving in to cravings”, A McNemar test was performed. Logistic regression analyzes were conducted to explore whether or not the following variables: age, BMI, Emotional eating, 5 personality traits (OCEAN), positive and negative affect, well-being (State of Mind ratio), had an effect on the probability of recognition of the proto-typical problem situations.

In order to answer Research Question 2, logistic regression analyzes were conducted to find out whether or not the type of coaching feedback had an effect on the probability of a respondent judging the coach’s feedback as good, or not good. A likelihood ratio test was conducted to examine the relation between the three coaching feedback conditions. Post-hoc pairwise comparisons (Wald tests with Holm correction) were conducted to test probabilities of differences among conditions.

## 3. Results

### 3.1. Research question 1

Are there predictors for recognition of the proto-typical problem situations for emotional eaters “experiencing cravings” and “after giving in to cravings”?

#### 3.1.1. Identification with problem situations “experiencing cravings” and “after giving in to cravings” (Q-1) presented by personas

A total of 79 respondents were asked whether they identified with resp. “experiencing cravings” and “after giving in to cravings” (entirely vs. not entirely). Comparing the paired samples proportions revealed that the respondents more often identified with “after giving in to cravings” (62%) than with “experiencing cravings” (47%) see Table 3. A McNemar test however showed that this difference was not statistically significant (*p* = 0.093). 55% of the respondents recognized themselves in both situations.

**Table 3:** Contingency table identification with “experiencing cravings” and “after giving in to cravings” (Q-1).

Q-1. Do you identify with “experiencing cravings”? & Do you identify with “after giving in to cravings”?		
	Identification with “experiencing cravings”	Identification with “after giving in to cravings”
Entirely	32 (47%)	49 (62%)
Not entirely	36 (53%)	30 (38%)
Total (*N*)	68 (100%)	79 (100%)

To find out whether or not the measured characteristics (BMI, age, emotional eating, positive and negative affect, well-being, and the personality characteristics openness, conscientiousness, extraversion, agreeableness, and neuroticism) had an effect on the probability of a respondent identifying with “experiencing cravings” or with “after giving in to cravings,” a logistic regression analysis was performed.

There was a significant positive association between BMI and identification with “experiencing cravings” (*χ*^2^ (1) = 9.47, *p* = 0.002; OR = 0.88, *p* = 0.005), with heavier women recognizing themselves more in the proto-typical problem situation of experiencing cravings. The same held true for the association of BMI with the identification of “after giving in to cravings” (*χ*^2^ (1) = 9.87, *p* = 0.002; OR = 0.87, *p* = 0.005).

Emotional eating was positively associated with both the identification with “experiencing cravings” and the identification with “after giving in to cravings” [respectively: (χ^2^ (1) = 6.06, *p* = 0.014; OR = 0.37, *p* = 0.023) and (χ^2^ (1) = 13.9, *p* < 0.001; OR = 0.19, *p* = 0.002)]. Women with high degrees of self reported emotional eating identified themselves more with both the identification with “experiencing cravings” and the identification with “after giving in to cravings” than women with lower degrees of self reported emotional eating.

Emotional stability (neuroticism) was only significantly and positively associated with the identification with “experiencing cravings”, but not with the identification with “after giving in to cravings” (respectively: *χ*^2^ (1) = 4.26, *p* = 0.039; OR = 1.66, *p* = 0.047, and *χ*^2^(1) = 1.29, *p* = 0.256). Women with self endorsed lower emotional stability (higher neuroticism) identified themselves more with the proto-typical problem situation “experiencing cravings”, than women with self endorsed higher emotional stability (lower neuroticism).

##### 3.1.1.1. In summary

Both the problem situations “experiencing cravings” and “after giving in to cravings” presented by two personas were recognized by participants. Higher levels of BMI were strongly associated with both the problem situations “experiencing cravings” and “after giving in to cravings.” Higher levels of emotional eating were strongly associated with recognition of both situations: “experiencing cravings” and “after giving in to cravings.” Low emotional stability (neuroticism) was strongly associated with “experiencing cravings.” For all other measured characteristics (age, positive and negative affect, well-being, and the personality characteristics openness, conscientiousness, extraversion, and agreeableness), no significant relationships were found.

### 3.2. Research question 2

What are the preferences for a specific coaching strategy with regards to typical problem situations for emotional eating?

#### 3.2.1. Problem situation “experiencing cravings” – opinion about the coach’s feedback (Q-2) and opinion on feedback coach to receive personally (Q-3)

To find out whether or not the type of coaching feedback (Condition: Validation vs. Focus-on-Change vs. Dialectical) had an effect on the probability of a respondent judging the coach’s feedback as good (as opposed to not good), a logistic regression analysis was performed.

Estimated probabilities of a positive opinion (Q-2 Opinion about the coach’s feedback) for all three conditions are presented in Table 4. In the Focus-on-Change coaching feedback condition the probability of a positive opinion on the coach’s feedback was lower than in the Validation and Dialectical coaching feedback conditions. As can be seen in Table 4, there are substantial differences between the three conditions in the estimated probabilities with relatively high probabilities in both the Validation and the Dialectical condition. The follow-up question (Q-3) was what the opinion of the women would have been if they personally had received this answer from the coach. Similar results as with Q-2 were revealed.

**Table 4:** “Experiencing cravings.” Effect of type of coaching feedback on the probabilities of a positive opinion (Q-2 and Q-3).

95% confidence interval								
coaching feedback	Prob.		SE		Lower		Upper	
	Q-2	Q-3	Q-2	Q-3	Q-2	Q-3	Q-2	Q-3
Validation	0.826	0.818	0.0790	0.0822	0.6177	0.604	0.933	0.930
Focus-on-Change	0.217	0.238	0.0860	0.0929	0.0935	0.103	0.428	0.460
Dialectical	0.826	0.905	0.0790	0.0640	0.6177	0.689	0.933	0.976

A likelihood ratio test showed that the differences between the three coaching feedback conditions were statistically significant for both Q-2 and Q-3 (respectively *χ*^2^ (2) = 24.8, *p* < 0.001, and *χ*^2^ (2) = 25.2, *p* < 0.001). Post-hoc pairwise comparisons (Wald tests with Holm correction) indicated that the difference between the Focus-on-Change and Dialectical conditions (OR = 17.1) and the Validation and Focus-on-Change conditions (OR = 30.4) were statistically significant for both Q-2 and Q-3 (respectively OR = 0.06, *p* < 0.001, and OR = 0.07, *p* < 0.001), but the difference between the Validation and Dialectical conditions was not [resp. OR = 1, *p* = 1.00, and OR = 2.11, *p* = 0.42].

The three groups (Validation, Focus-on-Change, and Dialectical) were distinct. The probability of a participant giving a positive rating to Validation was higher than to Focus-on-Change. The probability of giving a positive rating to Dialectical was higher than to Focus-on-Change.

##### 3.2.1.1. In summary

In the “experiencing cravings” condition, the women preferred the Validation and Dialectical coaching strategies. There was no difference in their preference if it was the coach’s feedback in general, or if it was directed to themselves personally.

#### 3.2.2. Problem situation “after giving in to cravings” – Opinion about the coach’s feedback (Q-2) and opinion on feedback coach to receive personally (Q-3)

Estimated probabilities of a positive opinion for all three conditions are presented in Table 5. In the Validation coaching feedback condition the probability of a positive opinion on the coach’s feedback was lower than in the Focus-on-Change and Dialectical coaching feedback conditions. As can be seen in Table 5, there were substantial differences between the three conditions in the estimated probabilities with a relatively high probability in the Focus-on-Change condition. The follow-up question (Q-3) was what the opinion of the women would have been if they personally had received this answer from the coach. Similar results as with Q-2 were revealed. An exception was the relatively high probability for the Dialectical condition in Q-3.

**Table 5:** “After giving in to cravings.” Effect of type of coaching feedback on the probabilities of a positive opinion (Q-2 and Q-3).

95% confidence interval								
Coaching feedback	Prob.		SE		Lower		Upper	
	Q-2	Q-3	Q-2	Q-3	Q-2	Q-3	Q-2	Q-3
Validation	0.192	0.360	0.077	0.0960	0.082	0.199	0.387	0.560
Focus-on-Change	0.609	0.609	0.102	0.1018	0.402	0.402	0.782	0.782
Dialectical	0.464	0.704	0.094	0.0879	0.292	0.510	0.646	0.844

A likelihood ratio test showed that the differences between the three coaching feedback conditions were statistically significant for both Q-2 and Q-3 (respectively χ^2^ (2) = 9.62, *p* = 0.008, and χ^2^ (2) = 6.61, *p* = 0.037). Post-hoc pairwise comparisons (Wald tests with Holm correction) indicated that for Q-2 the difference between the Validation and Focus-on-Change conditions [(OR = 6.54) was statistically significant *p* = 0.013], for and for Q-3 the differnce between Validation and Dialectical (OR = 4.22) was significant (*p* = 0.045).

The three groups (Validation, Focus-on-Change, and Dialectical) were distinct. The probability of a participant giving a positive rating to Validation is smaller than to Focus-on-Change. The probability of giving a positive rating to Dialectical is smaller than to Focus-on-Change.

The differences between the Validation and Dialectical conditions (OR = 3.64) and between the Focus-on-Change and Dialectical conditions (OR = 0.56) were both not statistically significant (respectively *p* = 0.078 and *p* = 0.306). The same held for differences between the Validation and Focus-on-Change conditions (OR = 2.76) and between the Focus-on-Change and Dialectical conditions (OR = 1.53) in Q-3. With, respectively, *p* = 0.177 and *p* = 0.481, they both were not statistically significant.

The probability of a participant giving a positive rating to Validation is lower than to Dialectical. In contrast, the probability that the participant will give a positive rating to Focus-on-Change is greater than to Dialectical.

##### 3.2.2.1. In summary

**Figure 1:**
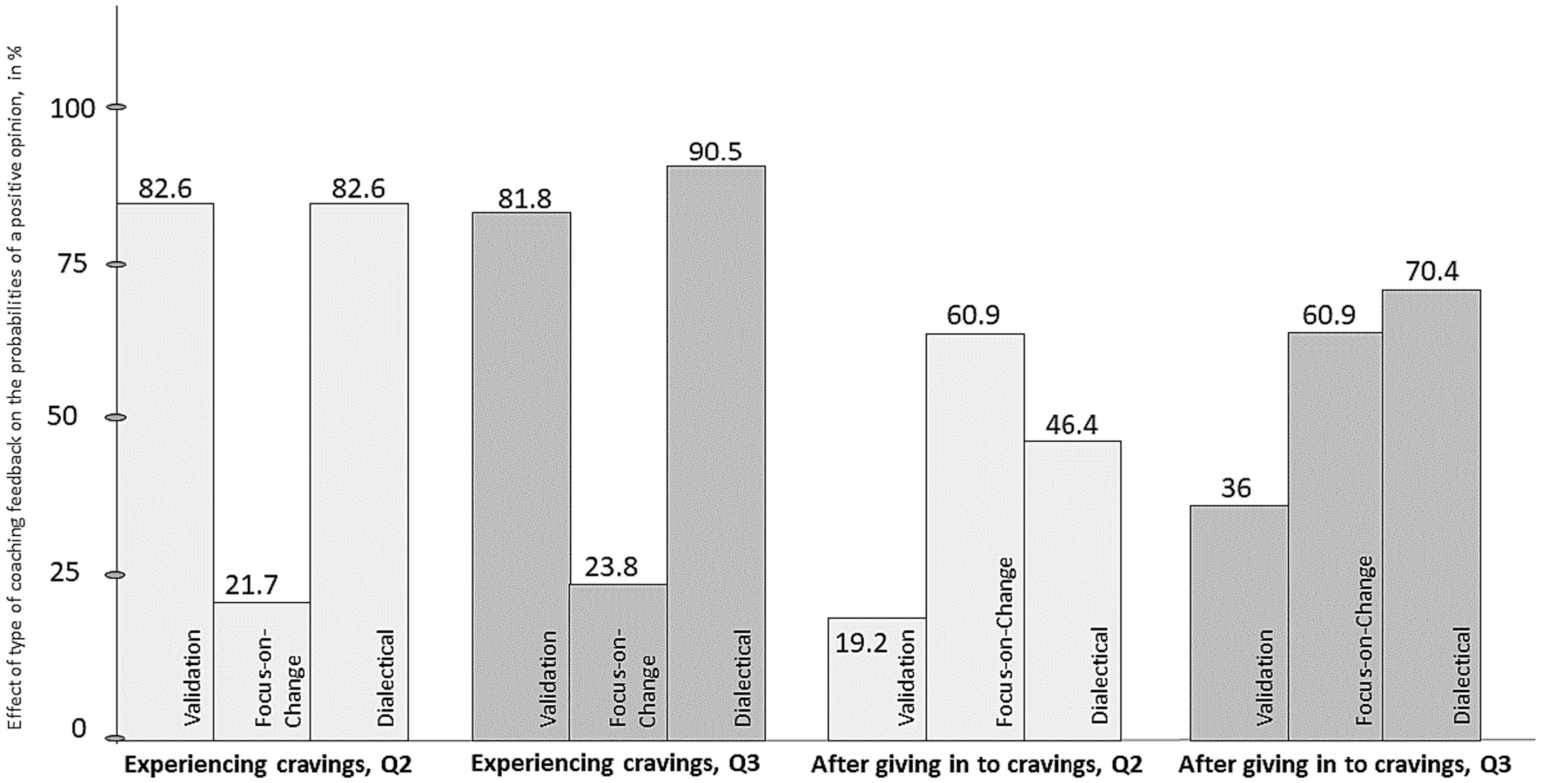
Opinion on feedback of the coach (Q-2, Q-3). Q-2 opinion on the feedback of the coach (to the personas), Q-3 opinion on the feedback of the coach if it was given to you personally.

In the “experiencing cravings” condition, the women preferred the Validation and Dialectical coaching strategies over that of Focus-on-Change. In the “after giving in to cravings” condition, they preferred the Focus-on-Change and Dialectical coaching strategies over that of Validation. There were differences in their preference whether it was the coach’s feedback in general (Q-2), or whether it was directed to themselves personally (Q-3) in Validation and Dialectical (Figure 1).

## 4. Discussion

*RQ1*: Are there predictors for identification with the proto-typical problem situations for emotional eaters “experiencing cravings” and “after giving in to cravings”?

To be in a position to meet the needs of prospective users of a personalized virtual coach, the present study examined whether respondents would identify with the presented problem situations.

It is crucial that participants and future users of the personalized virtual coach recognized themselves in one or both problem situations, because these two situations are the most essential situations, known by emotional eaters (Safer et al., 2009; Burton and Abbott, 2019; Dol et al., 2021). The fact that the participants recognized the presented problem situations well, contributes to the development of the virtual coach. With this, it can be accomplished that future users who are in trouble and consult the coach, immediately can be referred to appropriate coaching.

The majority of participants recognized the problem situations and women with a higher BMI and with a higher degree of self-reported Emotional Eating recognized themselves more often in both situations than women with lower BMI and lower self-reported Emotional Eating. This was also found for emotional instability in relation to “experiencing cravings”. Thus, the characteristics BMI, emotional eating and emotional stability can be considered as predictors in recognizing the problem situations “experiencing cravings” and “after giving in to cravings”. The added value of this knowledge may reside in the fact that future users of the virtual coach, who score on these characteristics, are better recognized and accommodated in their need for coaching. By taking such insights into account, coaching can be more tailor-made.

Considering the results, we can conclude that the personas were well recognized by the participants. In this context it can be noted that, though not statistically significant, the recognition for the personas was different from one another: 62% of them identified with the persona that represented the problem situation “after giving in to cravings”, compared to the 47% that identified with “experiencing cravings.”

The somewhat higher percentage of recognition may have arisen because at least five possible factors may have influenced the participants’ choices:

1. the appearance of the two different personas (the persona representing the problem situation “experiencing cravings”, Anita, is somewhat older and perhaps less appealing; the persona representing the problem situation “after giving in to cravings”, Lisanne, is young and pretty);
2. the description of the problem situations “Experiencing cravings” versus “After giving in to cravings”;
3. the described emotions in de problem situations; It is possible that “After giving in to cravings” appealed to more participants because the miserable feeling once the binge is over, the anger, the regret, the disappointment about one’s own behavior, shame may be more universal among emotional eaters than “Experiencing cravings,” because here stress is the described emotion that gives rise to cravings, while there are also other negative emotions such as fear, anger, annoyance and loneliness, which can be the prelude to experiencing and giving in to cravings (van Strien et al., 1986);
4. the appearance of the personas in terms of (cultural) background, education, and socioeconomic status. Anita, middle-aged, is a poorly educated woman. She has an anxious nature - she suffers from stress because of financial worries and concerns about her family. Lisanne, representing “after giving in to cravings”, is a well educated, good-looking young woman);
5. the order of presentation of the problem situations to the participants (first persona Lisanne and then Anita, while the order of “experiencing cravings” and ‘giving in to cravings’ is the other way around).

Participants may not have recognized the persona “experiencing cravings” as good as “after giving in to cravings” because the appearance of that persona was possibly less appealing. Participants looked at the picture and perhaps had their thoughts ready. A study by Salminen and colleagues showed that “[…] a smile enhances the perceived similarity with the persona, similar personas are more liked, and that likability increases the willingness to use a persona (Lau, 1982; Salminen et al., 2020). In follow-up research Salminen added to this that personas with happy pictures are perceived as more extroverted, agreeable, open, conscientious, and emotionally stable (Salminen et al., 2022).

In principle, both problem situations should be well recognized because the emotional eater experiences both problem situations. If an emotional eater suffers from cravings and gives in to them, then usually the phase “after giving in to cravings” is also lived through (Safer et al., 2009, 2018). An exception to this is when the emotional eater would have been able to resist the cravings.

However, it is not known which factors weighed more heavily for the participants because they were not surveyed on those aspects. It is therefore quite possible that the difference in appearance and presentation of the two personas could affect the internal validity of the study.

To increase the degree of persona recognition, it is important that preferences of participants are not affected by attributes other than those directly related to the specific characteristics of the emotional eater, and the proto-typical problem situations she can encounter.

*RQ2*: What are the preferences for a specific coaching strategy with regards to typical problem situations for emotional eating?

The objective of this study was to gain more knowledge about recognition of the given problem situations so that the virtual coach would be enabled to deliver the right coaching based on this information.

In the problem situation “experiencing cravings”, the women chose Validation and Dialectical over Focus-on-Change as the most appropriate coaching for them personally and as generally applicable coaching strategies. On the same questions regarding the problem situation “after giving in to cravings”, they preferred Focus-on-Change and Dialectical over Validation.

The coaching strategy of Validation seems to align with “experiencing cravings” but not as much for “after giving in to cravings”. A possible explanation for the fact that Validation may cause resentment in a person who just gave in to cravings is that she is ashamed of her behavior (giving in to cravings) and does not want her feelings and behavior to be ‘justified’ or even given a positive annotation.

Validation is valued in “experiencing cravings” but an equally large group preferred Dialectical. Validation is appreciated, but in combination with practical advice it has added value, because it could take the user further in her process of enduring cravings (Safer et al., 2009).

The Focus-on-change strategy seems to have possible interfaces with “after giving in to cravings” but not as much for “experiencing cravings”. A woman who just gave in to cravings needs practical advice. When she is still experiencing those cravings, she should be stopped from giving in to them, with the use of an instantaneous intervention. Perhaps the given advice was not the kind she was hoping for at that moment. She had already decided to leave the cookies in the cookie jar and had picked up her phone to contact the virtual coach. She did not need another confirmation from the coach. An advice might have been better, such as: “Anita, put on your coat and go out for a walk.” That could have helped her to temper the stress she was experiencing.

Dialectical feedback seemed appealing for women who were experiencing cravings. For those who already gave in to cravings it was preferred to some extent because they were not susceptible for the validating component of this coaching variety. Apparently they were only interested in practical tips with information on how to get her life back on track or how to prevent this from happening in the future.

So it seems that the virtual coach should make it clear to the client that Validation does not involve the act of giving in to cravings itself, but rather hinges on the client’s feelings of regret, or disgust or revulsion after giving in to cravings.

The Dialectical coaching strategy seems to get the best marks for both the problem situations. This style of coaching does well in the practitioner’s office (Linehan, 1993; Safer et al., 2009; Pederson, 2015), but seems to be applicable online as well. The Dialectical strategy should be more sharply emphasized to indicate that the Validation part of this coaching strategy has its focus on the client’s feelings of regret, or disgust or revulsion after giving in to cravings rather than the act of giving in to the cravings.

### 4.1. Strenghts and limitations of this study

Using personas is an efficient way to provide insights into how respondents relate to matters. They were generous in sharing their opinions on both the personas and the suggested coaching responses from the virtual coach (Dol et al., 2021). It might be advisable to include an additional round of verification to validate the personas with future users before conducting such a study. In that case, certain limitations of the current personas would have been revealed earlier, such as:

1. the personas differed from one another in terms of socioeconomic status, background and level of education,
2. it is not fully clear why participants did or did not recognize themselves in a persona,
3. the photos shown may have been too determinative.

These factors may have affected the study’s internal validity.

In future studies it is recommended that personas differ less from each other in person-related data such as socioeconomic status and background, but making the personas more comparable might have impact on the heterogeneity. Systematic methods have since been developed that underpin this problem (Holden et al., 2017; Klooster et al., 2022). By using the computational method silhouette clustering a better consistency of personas could be achieved.

When developing personas, it is important to strive for uniformity in personal profile and background to prevent participants from letting outward appearances influence their choices.

The strength of presenting personas using vignettes lies in the fact that it can be well applied when raising delicate topics. This promotes a sense of comfort among participants and encourages them to be open and approachable. The recognizability of the content with their everyday experiences creates a secure and credible context for them to reflect on the personas presented.

It is also important to note that the participants were a representative sample of individuals with heightened levels of emotional eating, which enhances the external validity of the results and suggests that the interpretations could be directly incorporated into virtual coaching practices for people dealing with emotional eating.

The percentages of recognition of the problem situations “experiencing cravings” and “after giving in to cravings” did not seem high. The explanation may be that we have imposed a strict standard in converting the 4 answer categories to 2 dichotomous variables. The initial answer categories that participants could choose from, were not articulated clearly enough. The response categories were as follows:

1) Yes, I can totally relate to L/A, 2) Yes, I can relate more or less, 3) No, I cannot relate very well, and 4) No, I cannot relate at all.

To keep the recoding beyond all reasonable doubt, only the first answer ‘Yes, I can totally relate to L/A’ is recoded to ‘entirely’. The other answers, namely ‘Yes, I can relate more or less’, ‘No, I cannot relate very well’ en ‘No, I cannot relate at all’ are recoded to ‘not entirely’. Working with such strict selections may have had ‘adverse’ effects on the results, but the result is beyond any doubt. Had response categories 1 and 2 been combined, 86.8 and 90.6% of participants, respectively, would have identified themselves “totally,” or “more or less,” in “experiencing cravings” and “after giving in to emotional eating.

Finally, a potential limitation may have been that participants were not asked if they knew of any other proto-typical problem situations.

## 5. Conclusion

Possible important predictors of identification with the presented problem situations are BMI, emotional eating and emotional stability. The relationships between these characteristics and the degree to which participants recognize themselves in the problem situations, may offer perspectives for developing a virtual coach application that resonates with the emotional eater’s experience.

The Dialectical coaching strategy apparently emerges as the most valued coaching strategy in both the proto-typical problem situations, namely “experiencing cravings” and “after giving in to cravings”. In contrast, Validation is rejected as an appropriate coaching strategy by a significant proportion of participants. The finding that a large proportion of the group rejects Validation as coaching strategy requires more insight into the emotional eater’s state of mind immediately after giving in to cravings.

The recognition of the personas provided more insight for the design of the virtual coach, but developing personas with more uniformity in personal profile and background, focussed on the characteristics of the typical problem situations of the emotional eater, could potentially contribute to more robustness of that design.

## Data availability statement

The raw data supporting the conclusions of this article will be made available by the authors, without undue reservation.

## Ethics statement

This study was approved by the ethics committee of the Faculty of Behavioural, Management and Social Sciences of the University of Twente (registration no. 18033) on 27 February 2018, and was conducted between 13 April and 13 May 2018. Written informed consent was obtained from the individuals for the publication of any potentially identifiable images or data included in this article.

## Author contributions

AD: Conceptualization, Formal analysis, Methodology, Writing – original draft. TS: Writing – review & editing. HV: Writing – review & editing. LG-P: Writing – review & editing. CB: Writing – review & editing.

## Footnotes

^1^For adults, WHO defines overweight and obesity as follows: overweight is a BMI greater than or equal to 25; and obesity is a BMI greater than or equal to 30. The abbreviation BMI stands for Body Mass Index.
