# Supplementary material for: Preferences for coaching strategies in a personalized virtual coach for emotional eaters: an explorative study
Source: Front Psychol. 2023 Nov 16;14:1260229. doi: 10.3389/fpsyg.2023.1260229 (PMC10687361; doi:10.3389/fpsyg.2023.1260229)
Supplement: Supplementary file 1 [file Presentation_1.pdf]

**Appendices**

Screenshots of the personas Anita and Lisanne as presented in Qualtrics to the participants. For translations, see below. The photos we used to personify the personas were purchased from Shutterstock.com)

**Figure A.** Persona Anita – respresenting the proto-typical problem situation ‘experiencing cravings’

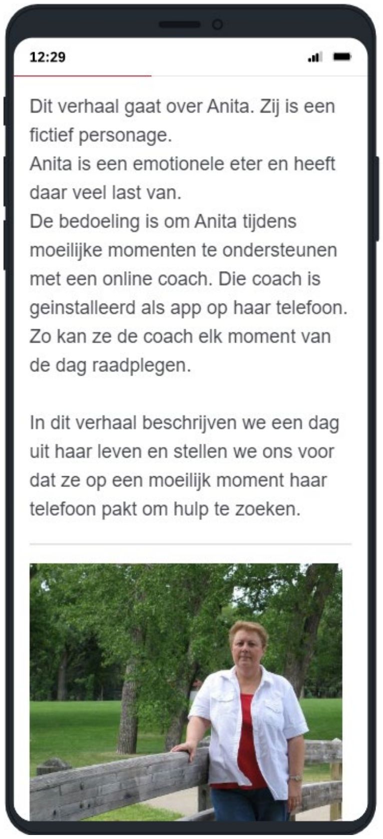


<u>Introduction vignette</u>

This story is about Anita. She is a fictional character. Anita is an emotional eater and suffers deeply from that.
The idea is to support Anita with an online coach during difficult moments. That coach is an app on her phone. So she can consult the coach any time of the day.
In this story, we describe a day in her life and imagine her grabbing her phone at a difficult moment to seek help.

Anita is 46 years old. She is married to Ad. They have two children, Melvin aged 19, and Leroy aged 16. Anita went to home economics school (LHNO). She works 3 days a week in home care. Other than that, she does housekeeping.Her hobbies are playing games on the computer and hiking (if Ad at least goes along). Star sign: Cancer.

Anita weighs 85 kilograms. She gains a few kilos every year. When tensions mount, Anita starts snacking. “Food provides a kind of comfort for me. When I feel stressed, I'm always snacking. That's really a problem, because because of this I've gained about 12 pounds in recent years.” Ad is a truck driver. The company he works for is having to downsize. He worries whether he will be allowed to stay. This causes a lot of stress in the family. Anita has known Ad since she was 14 years old. They got married when Anita was pregnant with Melvin.
Anita loves her job. She loves helping people. She has many contacts in the neighborhood and she regularly goes for coffee at the neighbors.

<u>A day in the life of Anita</u>
Anita hears that Melvin hasn't gotten out of bed yet and wakes him up so he won't be late for work. Melvin reacts grumpily, which upsets Anita. Leroy really needs to get up now, too. Anita wakes him up and tells him to go to school.
Anita fixes breakfast for the boys and starts the day with a cup of coffee herself. She is irritated because she still has to encourage the boys so much to get out of bed on time.
Anita goes shopping without eating breakfast. As a result, she buys many more goodies than she actually needs. Once back home, she cleans up the groceries and quickly eats a sandwich with jam. At the neighbor's house she drinks coffee. She also accepts a second slice of cake, because she is quite hungry.
Once home, she works through the house. The bathroom gets a major overhaul and she vacuums through the boys' rooms. She sits down on the couch for a while, just to relax. A cup of coffee, and a couple of sandwiches with peanut butter and jam for a late lunch.
At 4:30 Anita starts preparing dinner, chicken and noodles. Ad and the boys come home and they eat with the family. Ad tells Anita that a colleague's contract has not been renewed.
In the evening, the boys watch a soccer game. Anita plays a game on the laptop and pulls open a package of peanut butter cookies.
When Melvin throws some snacks in the fryer later in the evening, she finds it unsociable to say no, but actually she doesn't even really crave it.
....
The family goes to bed early. Ad always has to get up early. But Anita can't get to sleep. She keeps pondering about the dismissal of Ad's colleague. She sneaks down the stairs in the dark and sits down at the kitchen table with a cup of coffee and the cookie jar.
....
Anita grabs her phone and indicates in the app that she is about to indulge in cookies.

**Figure B.** Persona Lisanne - respresenting the proto-typical problem situation ‘after giving in to cravings’

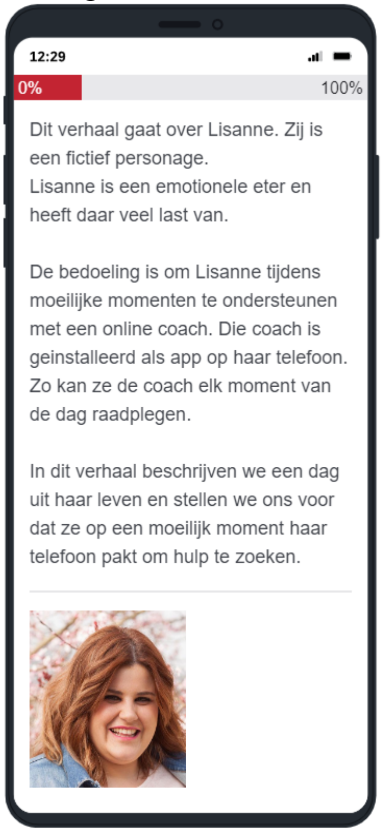


**Description persona Lisanne (Figure B)**

<u>Introduction vignette Lisanne</u>
This story is about Lisanne. She is a fictional character. Lisanne is an emotional eater and suffers deeply from that.
The idea is to support Lisanne with an online coach during difficult moments. That coach is an app  on her phone. So she can consult the coach any time of the day.
In this story, we describe a day in her life and imagine her grabbing her phone to seek help at a difficult moment.

Lisanne is 25 years old. She is single, living alone. She has lived together with Peter for two years. She has an MBO degree in commercial engineering and she works 5 days a week as an inside sales representative at a large real estate agency. Her hobbies are going out, and shopping. Sports: formerly korfball, but nothing at the moment. Starsign: pisces
Lisanne weighs 91 kilograms. She started dieting when she was 14 years old. Then she weighed 63 kilos. Lisanne feels insecure about her body. She is unable to feel the difference between hunger, cravings or emotion. She is unable to feel what she is really feeling. Once or twice a week she binges. Eating does relieve her for a while, but afterwards she feels horrible. She is disgusted with herself.

Lisanne is an only child. Her parents split up when she was 5 years old. She was very upset about that. Her mother was not very talkative, but expressed her feelings toward Lisanne by preparing her favorite food or bringing in treats, such as desserts, a bag of licorice, or tea and cookies.
Lisanne has a few friends, but she doesn't see them often nowadays. She knows Josje and Karin from her childhood, but they live on the other side of the country. Floor, an old classmate has just started a new relationship. Furthermore, she occasionally goes into town with colleague Esther. She used to play korfball fanatically, but nowadays she doesn't allow herself time for sports.

<u>A day in the life of Lisanne</u>
Lisanne gets out of bed too late - she set the alarm clock to snooze just a little too often.... So she skipped breakfast and took the car to work.
That morning she has a performance review at work. Her supervisor is quite pleased with her, but says she should take on more of a role in the sales team. She is quite flattered by her boss's words, but gets creeped out by the idea that she should be more visible in the group.
Back home Lisanne puts on her sweatpants and plops down on the couch. There is still a pile of laundry to be folded on the table. She turns on the TV, but she cannot find anything nice to watch.
When she thinks back to this morning's conversation, it really gets to her. She is overcome by feelings of insecurity. No way her colleagues like her! In her face they are behaving nicely, but in reality they think she is fat, and ugly, and stupid.
She should actually be cooking dinner, but she doesn't feel like it. There's nothing decent in the fridge anyway.... She shuffles to the kitchen, and browses all the cupboards...
....
Lisanne has just given in to a binge.
She went through all the cupboards and then ate a bag of potato chips, an already opened package of stale cookies, and two more sandwiches with a thick layer of hazelnut cocoa spread.
After the binge, she was disgusted with herself. Stupid girl! She let herself go all over again....
....
Lisanne grabs her phone and types that she has had a binge.

**Table 3**
*Overview of questions about the problem situations and three coaching strategies*

| Questions | Answering options (multiple choice (mc) and/or open text option | Recoded into dichotomies |
|---|---|---|
| Q-1. Do you identify with the persona Anita ('experiencing cravings') / persona Lisanne ('after giving in to cravings')? | mc<br>1 Yes, I can totally relate to L/A;<br>2 Yes, I can relate more or less;<br>3 No, I cannot relate very well;<br>4 No, I cannot relate at all. | 1 not entirely<br>2 entirely<br>(1=2) (2 thru 4=1) |
| Q-2. What is your opinion on the coaches' reaction to the problem of persona Anita ('experiencing cravings') / persona Lisanne ('after giving in to cravings')? | mc 2 + open text option<br>1 good;<br>2 not good, because... | 1 good<br>0 not good |
| Q-3. What would you think if you would got an answer like this yourself? | mc 5 + open text option<br>1 I find it helpful, that's what works for me;<br>2 I find it friendly, supportive, I feel understood;<br>3 I find it too confrontational;<br>4 I find it useless.<br>5 Other namely (explain): ... | 1 positive response<br>0 negative response<br>(1=1) (2=1) (3=0) (4=0) (5=SYSMIS) |

**Table 4**
*Overview of coaches' responses*

| problem situation / condition | Experiencing cravings: 'Anita grabs her phone and indicates in the app that she is about to overindulge in cookies.' | After giving in to cravings: 'Lisanne grabs her phone and types that she had a binge.' |
|---|---|---|
| action: | The coach responds to Anita: | The coach responds to Lisanne: |
| Validation | "Hi Anita, how good of you to leave the cookies. I know how hard that is. You have every reason to be proud of yourself. " | "Hi Lisanne, annoying that you had a binge. I understand that after such a day as today you did have a need for food and comfort." |
| Focus-on-change | "Hi Anita, try to hold on to the feeling you have now and remember that when you are about to snack again." | "Hi Lisanne, Good that you are consulting the app! Don't be angry with yourself, it takes time to change. Give yourself that time. What could you do right now to get rid of that annoying feeling and get back on track?" |
| Dialectical | "Hi Anita, how good of you to leave the cookies. I know how hard that is. | "Hi Lisanne, too bad you that you had a binge. After a day like today, you do have |

| | | |
|---|---|---|
| | You have every reason to be proud of yourself. But try to hold on to the feeling you have now and remember that when you are about to snack again." | a need for food and comfort. Don't be angry to yourself. The challenge now is to take good care of yourself. What could you do right now to get rid of that annoying feeling and get back on track?" |

For the purpose of inter-rater reliability recoding of the textual responses of participants in the answer category "Other, namely..." to "1 positive" and "0 negative" (Q-3) was carried out in cooperation with a fellow researcher (MvL). The items were discussed one by one. Decision-making was carried out on the basis of the consensus model.
